# Phosphorylation of S6RP in peritubular capillaries of kidney grafts and circulating HLA donor-specific antibodies

**DOI:** 10.3389/fmed.2022.988080

**Published:** 2022-10-18

**Authors:** Dalia Raïch-Regué, Javier Gimeno, Laura Llinàs-Mallol, Silvia Menéndez, David Benito, Dolores Redondo, M. José Pérez-Sáez, Marta Riera, Elaine F. Reed, Julio Pascual, Marta Crespo

**Affiliations:** ^1^Hospital del Mar Medical Research Institute (IMIM), Barcelona, Spain; ^2^Department of Nephrology, Hospital del Mar, Barcelona, Spain; ^3^Department of Pathology, Hospital del Mar, Barcelona, Spain; ^4^Department of Pathology and Laboratory Medicine, David Geffen School of Medicine, University of California, Los Angeles, Los Angeles, CA, United States

**Keywords:** transplantation—kidney, donor specific antibodies, mammalian target of rapamycin (mTOR), phosphorylation, peritubular capillaries, ribosomal protein S6 (S6RP), antibody mediated allograft rejection

## Abstract

Antibody-mediated rejection (ABMR) caused by donor-specific HLA-antibodies (DSA) is a mediator of allograft loss after kidney transplantation (KT). DSA can activate microvascular endothelium damage through the mTOR pathway. In this study we assessed the mTOR pathway activation by DSA in KT with ABMR (ABMR + DSA+) compared to controls (ABMR−DSA−), biopsies with ABMR changes without DSA (ABMR + DSA−) and DSA without ABMR changes (ABMR−DSA+), and the potential modulation by mTOR inhibitors (mTORi). We evaluated 97 biopsies: 31 ABMR + DSA+, 33 controls ABMR-DSA−, 16 ABMR + DSA−, and 17 ABMR-DSA+ cases. Regarding immunosuppression of full ABMR + DSA+ and controls, 21 biopsies were performed under mTORi treatment (11 of them ABMR + DSA+ cases) and 43 without mTORi (20 of them ABMR + DSA+) so as to explore its effect on the mTOR pathway. Biopsies were stained for C4d, Ki67, and phosphorylated (p) S6RP, ERK, and mTOR by immunohistochemistry. Labeling was graded according to peritubular capillary staining. ABMR biopsies showed significantly higher C4d, p-S6RP, and Ki67 staining in peritubular capillaries (PTC) compared to controls, and light differences in p-ERK or p-mTOR. mTORi treatment did not modify p-S6RP, p-mTOR, and p-ERK staining. Diffuse p-S6RP in PTC in the biopsies significantly associated with circulating HLA-DSA independently of graft rejection, and with worse death-censored graft survival. These findings suggest that activation of endothelium through the mTOR pathway evidence different mechanisms of damage in ABMR + DSA+ and ABMR + DSA− despite similar histological injury.

## Introduction

Antibody-mediated rejection (ABMR) is one of the leading causes of renal allograft loss (1). Antibodies against human leukocyte antigens (HLA) of the donor (DSA) are associated with poor allograft survival, frequently preceding this type of graft rejection (2, 3). ABMR is characterized by microvascular lesions in the form of inflammation and/or tissue remodeling in the presence of HLA-DSA (1, 4). Circulating HLA-DSA bind to graft endothelial cells and exert multiple effector functions that may produce damage, such as immune cell recruitment, complement activation or transduction of intracellular signals, leading to proliferation of graft vasculature (5). In fact, subsequent chronic transplant glomerulopathy and peritubular capillary basement membrane multilayering are diagnostic criteria for chronic ABMR. Understanding and documentation of these mechanisms of damage is paramount to diagnose, prevent and treat injury produced in ABMR.

Activation of complement in the form of C4d deposits in peritubular capillaries (PTC) was originally proposed as ABMR marker in renal transplant biopsies (6). Initially, C4d positivity was incorporated in the definition of ABMR in the Banff Classification (7), showing strong correlation with the presence of DSA (8). However, not all ABMR cases are C4d positive, as recognized by the Banff classification since 2013 (9), and there are also C4d-positive biopsies without evidence of rejection (10). Therefore, the implementation of new molecular diagnostic markers for ABMR may improve diagnosis and management of ABMR.

Vascular injury in ABMR is accompanied by endothelial cell activation (11). Microvascular endothelial cells express HLA constitutively (12), and expression of HLA class-II increases after transplantation (13). HLA class-II DSA are more strongly associated with microvascular injury and chronic endothelial lesions than HLA class-I DSA (14). Binding of anti-HLA class-I and –II antibodies to microvascular endothelial cells allows the recruitment of Integrin-β4 and other not well defined molecules, which transduce cytoplasmatic signals through the mammalian target of rapamycin (mTOR) pathway (15–17). mTOR is a protein kinase that forms two molecular complexes, 1 and 2, with distinct functional capacities. mTOR also integrates the input from other extracellular signals, such as insulin, growth factors, amino acids, and oxygen (18), and is a key regulator of cell growth, cell proliferation and survival, protein synthesis and autophagy (19). Indeed, mTOR was described to play important roles in different tissues, such as liver or brain, and to be dysregulated in human diseases like cancer or diabetes (20). In this line, up-regulated mTOR activity was found in active injury in both native and transplanted human kidneys (21). Furthermore, phosphorylation of the mTOR pathway proteins S6RP and 70S6K has been proposed as new ABMR markers on transplanted hearts (22, 23). Inhibitors of mTOR (mTORi; rapamycin or everolimus) have been used for two decades to prevent organ transplant rejection. *In vitro*, everolimus has shown to inhibit anti-HLA antibody-mediated signaling, migration and proliferation of endothelial cells (15, 24).

We hypothesized that detection of phosphorylated (p)-mTOR and its downstream signals S6RP and ERK in kidney allografts may be useful as diagnostic biomarkers of ABMR in the presence of circulating HLA-DSA, and the use of mTORi may modulate the pathway *in vivo*. Therefore, we evaluated the activation signals of the mTOR pathway: p-mTOR, p-S6RP and p-ERK, as well as microvascular endothelial proliferation in kidney allografts with histological criteria of ABMR with and without HLA-DSA, DSA without ABMR, and with normal samples. We also assessed the potential modulation of the mTOR pathway by mTORi immunosuppression *in vivo*.

## Materials and methods

### Patient study population

We included 97 kidney transplant (KT) recipients with graft-biopsies performed between 2011 and 2015, around 24 months post-transplantation [IQR 14–56]. First, the study group comprised 31 patients diagnosed of ABMR with HLA-DSA (ABMR^+^DSA^+^), and 33 age-matched recipients with normal biopsies (ABMR^–^DSA^–^) according to (25). Of them, 21 patients received treatment with mTORi (20 Everolimus, 1 Rapamycin) at the time of biopsy (11 ABMR + DSA+) and 43 did not (20 ABMR + DSA+). Second, we included patients with an ABMR histological phenotype who lacked HLA-DSA (ABMR + DSA−, *n* = 16) and patients with circulating HLA-DSA without ABMR histological diagnosis (ABMR−DSA+, *n* = 17). Patient characteristics and clinical data are shown in Tables 1–4 and Supplementary Table 1. The study was approved by the CEIC Parc de Salut Mar Ethical Research Board (2018/7873I) and all patients signed informed consents. The clinical and research activities being reported are consistent with the Principles of the Declaration of Istanbul and the Declaration of Helsinki.

**TABLE 1:** Main demographic and clinical characteristics of patients with ABMR and HLA-DSA (ABMR^+^DSA^+^) and without ABMR and HLA-DSA (ABMR^–^ DSA^–^).

	ABMR^–^ DSA^–^ (*n* = 33)	ABMR^+^ DSA^+^ (*n* = 31)	*P*-value
Recipient age (years) [mean (SD)]	49.6 (11.5)	45.6 (16.0)	0.26
Recipient gender (female) [*n* (%)]	13 (39.4%)	15 (48.4%)	0.62
Type of donor (deceased) [*n* (%)]	25 (75.8%)	29 (93.5%)	0.083
Donor age (years) [mean (SD)]	51.8 (12.7)	44.2 (17.6)	0.056
Re-transplantation [*n* (%)]	1 (3.0%)	10 (32.3%)	**0.002**
HLA-DSA^+^ at transplantation [n/total (%)]*	1/32 (3.1%)	11/27 (40.7%)	**< 0.001**
Antilymphocyte induction [*n* (%)]	3 (9.1%)	6 (19.4%)	0.22
**Immunosuppression at transplantation [ *n* (%)]**			
Calcineurin inhibitors	33 (100%)	33 (100%)	1.00
Mycophenolate mofetil	26 (78.8%)	27 (87.1%)	0.51
mTOR inhibitor	7 (21.2%)	2 (6.5%)	0.15
Biopsy-proven acute rejection <3 months after transplantation [*n* (%)]	2 (6.5%)	5 (16.1%)	0.25
**At biopsy**			
Biopsy time after KT (months) [median (IQR)]	38.5 (16–41)	36.7 (14–98)	0.39
Indication biopsy [*n* (%)]	4 (12.1%)	15 (48.4%)	**0.002**
**Immunosuppression [*n* (%)]**			
Prednisone	30 (90.9%)	25 (80.6%)	0.30
Calcineurin inhibitors	29 (87.9%)	21 (67.7%)	0.071
Mycophenolate	26 (78.9%)	28 (90.3%)	0.31
mTOR inhibitors	10 (30.3%)	11 (35.5%)	0.79
**Graft function**			
Serum creatinine [mg/dl, mean (SD)]	1.3 (0.4)	1.9 (0.9)	**0.002**
eGFR [ml/min, mean (SD)]	64.1 (25.4)	46.6 (22.8)	**0.005**
Pr/Cr [mg/g, median (IQR)]	116.2 (77–187)	389.0 (167–994)	**< 0.001**

ABMR, antibody-mediated rejection; DSA, HLA donor specific antibodies; eGFR, estimated glomerular filtration rate; IQR, interquartile range; KT, kidney transplantation; Pr/Cr, urinary protein to creatinine ratio; SD, standard deviation. *From 59 patients with pre-transplant SAB assay performed. A significant *p*-value is indicated by a bold value.

**TABLE 2:** Comparison of the histopathologic lesions in the biopsy of patients with ABMR and HLA-DSA (ABMR^+^DSA^+^) and without ABMR and HLA-DSA (ABMR^–^ DSA^–^).

	ABMR^–^ DSA^–^ (*n* = 33)	ABMR^+^ DSA^+^ (*n* = 31)	*P*-value
Percentage of glomerulosclerosis [mean (SD)]	17.2% (3.0)	13.9% (2.9)	0.44

Glomerulitis (g ≥ 1) (yes, %)	2 (6.1)	24 (77.4)	**< 0.001**
g0	31 (93.9)	7 (22.6)	
g1	2 (6.1)	11 (35.5)	
g2	0 (0)	9 (29.0)	
g3	0 (0)	4 (12.9)	

Peritubular capilaritis (PTC ≥ 1) (yes, %)	1 (3.0)	25 (80.6)	**< 0.001**
ptc0	32 (97.0)	6 (19.4)	
ptc1	1 (3.0)	18 (58.0)	
ptc2	0 (0)	7 (22.6)	
ptc3	0 (0)	0 (0)	

Microvascular inflammation (g + PTC ≥ 2) (yes, %)	0 (0)	25 (80.6)	**< 0.001**

C4d positivity (C4d ≥ 1) (yes, %)*	0 (0)	11 (36.7)	**< 0.001**
C4d0	33 (100)	15 (50.0)	
C4d1	0 (0)	4 (13.3)	
C4d2	0 (0)	3 (10.0)	
C4d3	0 (0)	8 (26.7)	

Chronic transplant glomerulopathy (yes, %)	0 (0)	16 (51.6)	**< 0.001**
EMCTG or PTCML (yes, %)^#^	0 (0)	24 (82.8)	**< 0.001**
Arteriolar hialinosis (ah ≥ 1) (yes, %)*	15 (45.5)	15 (50.0)	0.80
Arterial intimal fibrosis (cv ≥ 1) (yes, %)^$^	13 (39.4)	13 (46.4)	0.61
Interstitial fibrosis (ci ≥ 1) (yes, %)	24 (72.7)	28 (90.3)	0.11
Tubular atrophy (ct ≥ 1) (yes, %)	25 (75.8)	26 (83.9)	0.54
Tubulitis (*t* ≥ 1) (yes, %)	0 (0)	7 (22.6)	**0.004**
Interstitial inflammation (*i* ≥ 1) (yes, %)	0 (0)	5 (16.1)	**0.022**
Intimal arteritis (*v* ≥ 1) (yes, %)**	0 (0)	1 (3.2)	0.44

ABMR, antibody-mediated rejection; CTG, chronic transplant glomerulopathy; DSA, HLA donor specific antibodies; EM, electron microscopy; PTCML, peritubular capillary multilayering; SD, standard deviation. *From 63 biopsies. ^#^From 46 biopsies with EM study. ^$^From 61 biopsies. **From 59 biopsies. A significant *p*-value is indicated by a bold value.

**TABLE 3:** Main demographic and clinical characteristics of patients with ABMR and DSA compared to ABMR without HLA-DSA (left) and to non-ABMR with HLA-DSA (right).

	ABMR^+^ DSA^+^ (*n* = 31)	ABMR^+^ DSA^–^ (*n* = 16)	*P*-value	ABMR^–^ DSA^+^ (*n* = 17)	*P*-value vs. ABMR^+^ DSA^+^
**AT TRANSPLANTATION**					
Recipient age (years) [mean (SD)]	45.6 (16.0)	51.3 (15.9)	0.25	49.1 (17.9)	0.49
Recipient gender (female) [*n* (%)]	15 (48.4%)	8 (50.0%)	1.00	7 (41.2%)	0.77
Type of donor (deceased) [*n* (%)]	29 (93.5%)	12 (75.0%)	0.16	17 (100%)	0.53
Donor age (years) [mean (SD)]	44.2 (17.6)	51.8 (13.5)	0.14	48.9 (20.2)	0.41
HLA-DSA^+^ at transplantation [n/total (%)]*	11/27 (40.7%)	1/12 (8.3%)	0.090	6/16 (37.5%)	1.00
Thymoglobulin induction [*n* (%)]	6 (19.4%)	4 (25.0%)	0.38	6 (35.3%)	0.32
**AT BIOPSY**					
Biopsy time after KT (months) [median (IQR)]	36.7 (14–98)	15.2 (8–80)	0.27	18.5 (14–64)	0.50
Immunosuppression at biopsy [ *n* (%)]					
Prednisone	25 (80.6%)	13 (81.3%)	1.00	14 (82.4%)	1.00
Calcineurin inhibitors	21 (67.7%)	15 (93.8%)	0.070	15 (88.2%)	0.17
Mycophenolate	28 (90.3%)	11 (68.8%)	0.10	16 (94.4%)	1.00
mTOR inhibitors	11 (35.5%)	3 (18.8%)	0.32	3 (17.6%)	0.32
ABMR characteristics in biopsy [ *n* (%)]				N/A	N/A
Microvascular inflammation (g + PTC ≥ 2)	25 (80.6%)	14 (87.5%)	0.70		
Glomerulitis (g ≥ 1)	24 (77.4%)	12 (75.0%)	1.00		
PTC (PTC ≥ 1)	25 (80.6%)	9 (56.3%)	0.096		
C4d deposits (C4d ≥ 2)	11 (35.5%)	5 (31.3%)	0.76		
Chronic transplant glomerulopathy [yes (%)]	16 (51.6%)	6 (37.5%)	0.54		
EM CTG or PTCML [yes/total (%)]**	24/29 (82.8%)	5/9 (55.6%)	0.17		
Serum creatinine [mg/dl, mean (SD)]	1.9 (0.9)	2.3 (1.8)	0.21	1.3 (0.4)	**0.024**
eGFR [ml/min, mean (SD)]	46.6 (22.8)	40.6 (22.7)	0.39	58.1 (16.1)	0.074
Pr/Cr [mg/g, median (IQR)]	389.0 (167–994)	604 (144–910)	0.98	155.9 (111–244)	**0.004**

ABMR, antibody-mediated rejection; CTG, chronic transplant glomerulopathy; DSA, HLA donor specific antibodies; eGFR, estimated glomerular filtration rate; EM, electron microscopy; IQR, interquartile range; KT, kidney transplantation; Pr/Cr, urinary protein to creatinine ratio; PTC, peritubular capillaries; PTCML, peritubular capillary multilayering; SD, standard deviation. *From 55 patients with pre-transplant SAB assay performed; **From 38 patients with EM study. A significant *p*-value is indicated by a bold value.

**TABLE 4:** Clinical follow-up of patients with ABMR and DSA compared to ABMR without HLA-DSA (left) and to non-ABMR with HLA-DSA (right).

	ABMR^+^ DSA^+^ (*n* = 31)	ABMR^+^ DSA^–^ (*n* = 16)	*P*-value	ABMR^–^ DSA^+^ (*n* = 17)	*P*-value vs. ABMR^+^ DSA^+^
1-year follow-up post-biopsy	(*n* = 26)	(*n* = 14)	0.45	(*n* = 17)	**0.004**
Serum creatinine [mg/dl, mean (SD)]	1.8 (0.7)	2.0 (0.9)	0.55	1.3 (0.3)	**0.040**
eGFR [ml/min, mean (SD)]	45.2 (23.1)	40.5 (24.6)	0.88	57.0 (13.2)	**0.042**
Pr/Cr [mg/g, median (IQR)]	258.0 (80–946)	609.5 (73–1077)		123.0 (70–214)	
4–5 years follow-up post-biopsy	(*n* = 19)	(*n* = 9)	0.71	(*n* = 10)	**0.043**
Serum creatinine [mg/dl, mean (SD)]	2.0 (1.1)	1.8 (0.6)	0.51	1.4 (0.4)	0.17
eGFR [ml/min, mean (SD)]	43.7 (22.7)	37.9 (17.8)	0.19	54.7 (13.8)	**0.002**
Pr/Cr [mg/g, median (IQR)]	521.5 (122–1527)	202.0 (84–474)		70.0 (58–88)	
Time of follow-up after biopsy (months) [median (IQR)]	69.9 (25–95)	50.3 (26–58)	0.19	70.8 (39–91)	0.87
Death censored-graft loss [*n* (%)]	13/31 (41.9%)	6/16 (37.5%)	1.00	0/17 (0%)	**0.002**
Graft loss including death [*n* (%)]	16/31 (51.6%)	9/16 (56.3%)	1.00	2/17 (11.8%)	**0.011**
Subsequent biopsy with ABMR [yes (%)]^#^	(*n* = 12) 11/12 (91.7%)	(*n* = 6) 4/6 (66.7%)	0.25	(*n* = 6) 2/6 (33.3%)	**0.022**

ABMR, antibody-mediated rejection; DSA, HLA donor specific antibodies; eGFR, estimated glomerular filtration rate; IQR, interquartile range; Pr/Cr, urinary protein to creatinine ratio; SD, standard deviation. ^#^From 24 patients with a subsequent biopsy realized. A significant *p*-value is indicated by a bold value.

### Immunohistochemistry and grading of phosphorylated proteins

Immunohistochemistry was performed on 3-μm paraffin allograft-biopsy sections, placed on plus-charged glass slides. After deparaffinization, heat antigen retrieval was performed in a pH = 9 EDTA-buffered solution (Dako, CA, United States). Endogenous peroxidase was quenched. Primary antibodies were incubated for 1 h at room temperature: anti-phospho-mTOR at Ser2448 (clone 49F9) rabbit mAb at 1:100, anti-phospho-S6 ribosomal protein at Ser235/236 (clone 91B2) rabbit mAb at 1:80, and anti-phospho-ERK1/2 (clone 20G11) rabbit mAb at 1:150, all from Cell Signaling Technology (Netherlands), and anti-Ki67 (clone MIB-1; Agilent, Dako) mAb at 1:100. Antigen–antibody reaction was detected by an anti-mouse/rabbit Ig–dextran-polymer coupled with peroxidase (Flex+, Dako). Sections were visualized with 3,3’-diaminobenzidine and counterstained with hematoxylin. Staining was performed on a Dako Autostainer platform. Protein expression was blindly assessed by one pathologist (J.G.).

Peritubular capillaries labeling was graded according to the staining intensity scale: 0 = none; 1 = rare; 2 = focal; 3 = multifocal/diffuse. A score ≥ 1 was considered positive for p-mTOR, and a score = 3 was considered positive for p-ERK and p-S6RP, according to the staining sensitivity of each marker. Due to limitations on the amount of sample, staining of p-S6RP was performed in 95 samples, p-ERK in 61 samples, p-mTOR in 61 samples, and Ki67 in 40 samples from the initial cohort.

C4d immunostaining was performed on 3-μm −80°C frozen biopsy sections. Antigen retrieval was performed with Cell Conditioning 1 (Roche, United States) for 8min. Primary anti-C4d polyclonal antibody (Cell Marque, CA, United States) was incubated for 28 min at room temperature and performed on a Benchmark XT platform (Roche). Sections were visualized using the Roche Optiview Kit. C4d staining was classified as minimally, focally or diffusely positive according to Banff classification.

### Evaluation of anti-human leukocyte antigens antibodies

Sera samples were collected contemporaneously to the biopsy (0, [−15, 34.5] days) and stored at −80°C until their analysis. Antibody testing was performed using Luminex^®^ HLA Single Antigen Bead assays (LABScreen, One Lambda, CA, United States) with a normalized mean fluorescence intensity (MFI) of 1000 as a cutoff for identification of a relevant HLA antibody. Donor HLA antibody specificity was ascribed considering donor HLA A, B, DRB1 and some C and DQB typing. Antibodies against some C or DQ antigens were assigned considering linkage disequilibrium.

### Statistical analysis

Comparisons between normally distributed variables were carried out using Student’s *t*-test, and non-parametric variables were analyzed with *U* Mann-Whitney test or with Kruskal–Wallis test. Normal distribution of continuous variables was tested with Kolgorov-Smirnoff and Shapiro-Wilk tests. Chi-squared or Fisher’s exact tests were used for dichotomous variables. AUC (Area under the curve) values for “C4d,” “p-S6RP,” and “C4d and p-S6RP” were compared using the DeLong test for correlated AUC, in order to determine the best test in terms of diagnostic performance. A *p* < 0.05 was considered statistically significant. Statistical analysis was performed using SPSS^®^ v.22.0 (IBM Corp., New York, United States).

### Data availability

The data that support the findings of this study are available from the corresponding author upon reasonable request.

## Results

### Patients’ demographic and clinical characteristics

For this study, first we evaluated 64 patients, 31 had a biopsy with ABMR diagnosis and circulating HLA-DSA (ABMR^+^DSA^+^), and 33 with normal biopsies (ABMR^–^DSA^–^) were selected as controls. Baseline characteristics were similar in both groups (Table 1). Almost half of the ABMR^+^DSA^+^ biopsies were performed for indication, significantly more than ABMR^–^DSA^–^, and ABMR^+^DSA+ showed worse serum creatinine and glomerular filtration rate (eGFR), and higher protein-creatinine ratio (Pr/Cr) at biopsy than ABMR^–^DSA^–^ (Table 1). As expected, ABMR^+^DSA^+^ biopsies presented worse histopathologic lesions compared to ABMR^–^DSA^–^ biopsies (Table 2). Clinical characteristics of patients treated or not with mTORi were similar, except for the proportion of patients receiving calcineurin inhibitors and/or mycophenolate (Supplementary Table 1).

Then, we included a group of patients with ABMR changes without HLA-DSA (ABMR^+^DSA^–^, *n* = 16) and a group with HLA-DSA but no ABMR changes (ABMR^–^DSA^+^, *n* = 17) in the biopsies. At baseline, there were no significant differences among groups (Table 3). At biopsy, patients with ABMR^–^DSA^+^ showed a significantly lower serum creatinine and lower protein-creatinine ratio (Pr/Cr) compared to ABMR^+^DSA^+^.

Patient follow-up at 1 and 4/5 years is shown in Table 4. Interestingly, we observed that ABMR^–^DSA^+^ patients had better graft function and lower proteinuria, less frequently a subsequent biopsy with ABMR, and less graft failure in comparison with 41.9% censored-graft loss in ABMR^+^DSA^+^ group. No significant differences were found when comparing ABMR^+^DSA^+^ with ABMR^+^DSA^–^ during the follow-up. Data on death-censored graft survival is consistent with these results, showing a significant difference on graft survival between ABMR- and ABMR+ cases regardless of DSA detection (Supplementary Figure 1).

Considering all ABMR cases, with and without DSA (*n* = 47), we identified 2 mixed rejections, both of them corresponding to TCMR grade IIA (v1 lesions), and 8 cases with borderline changes concomitant to the ABMR histology.

### Phospho-S6 ribosomal protein at Ser235/236 staining in peritubular capillaries is increased in antibody-mediated rejection biopsies

**FIGURE 1:**
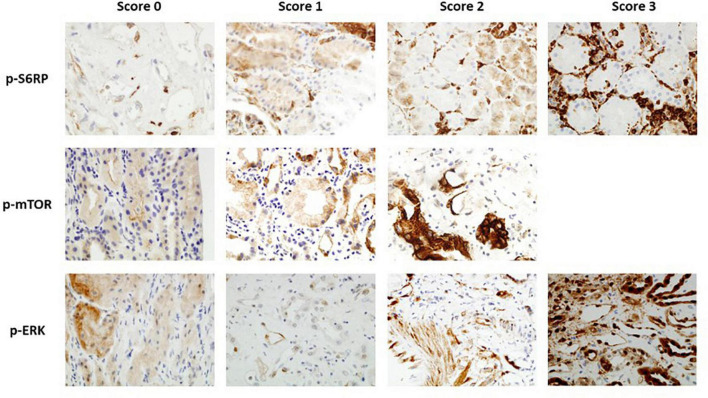
Immunohistochemistry staining of p-S6RP, p-ERK, and p-mTOR in kidney transplant biopsies (40×). Representative images of different staining scores for each protein are shown.

Detection of p-S6RP, p-ERK, and p-mTOR in PTC (Figure 1) was scored as previously described (23). P-S6RP and P-ERK positive cases showed a granular cytoplasmic staining in endothelial cells, with some cases displaying a similar staining pattern in glomerular endothelial cells and epithelial tubular cells. P-mTOR staining was also granular and cytoplasmic in PTC endothelium, less intense than the other markers, and was found in some biopsies in epithelial tubular cells with strong apical and membranous staining. Due to limitations on the amount of sample, staining of p-S6RP was performed in 95 samples, p-ERK in 61 samples, p-mTOR in 61 samples, and Ki67 in 40 samples.

**FIGURE 2:**
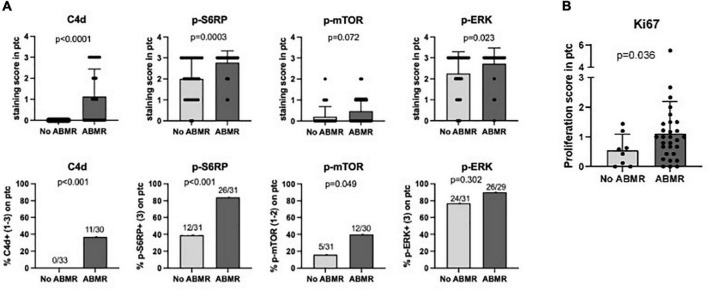
Staining of p-S6RP in peritubular capillaries is increased in ABMR biopsies. **(A)** Staining score (upper graphs) and percentage of positive staining (lower graphs) of C4d, p-S6RP, p-ERK, and p-mTOR in PTC of renal allografts biopsies with ABMR + DSA+ (*n* = 31) compared to ABMR–DSA– (*n* = 33). Mann Whitney and Fisher exact tests were used to assess significant differences between these two groups. **(B)** Staining of the proliferation marker Ki67 was analyzed in biopsies with and without ABMR. Plots show the means (bars), standard deviation (error bars), and individual values (dots) for each sample. Mann Whitney and Fisher exact tests were used to assess significant differences between groups.

We analyzed whether endothelial positive staining of p-S6RP, p-ERK or p-mTOR and C4d in PTC of renal allografts associated with ABMR^+^DSA^+^ compared to ABMR^–^DSA^–^. As expected, C4d was significantly increased in biopsies with ABMR compared to control biopsies (*p* < 0.0001; Figure 2A). Interestingly, staining of p-S6RP was significantly increased in ABMR^+^DSA^+^ biopsies compared to ABMR^–^DSA^–^ biopsies (*p* = 0.0003; Figure 2A). There was a slight difference between groups regarding the staining of p-ERK (*p* = 0.023) and no difference with p-mTOR (*p* = 0.072) (Figure 2A).

Overall, these results suggest that positive staining of p-S6RP in PTC of renal biopsies is associated with the interaction of DSA with endothelium. Given that HLA-DSA have been reported to activate proliferation of endothelial cells, staining with the proliferation marker Ki67 was analyzed in biopsies with and without ABMR. Accordingly, ABMR^+^DSA^+^ biopsies showed a significantly increased Ki67 staining score in endothelium of PTC compared with ABMR^–^DSA^–^ biopsies (*p* = 0.036, Figure 2B).

**FIGURE 3:**
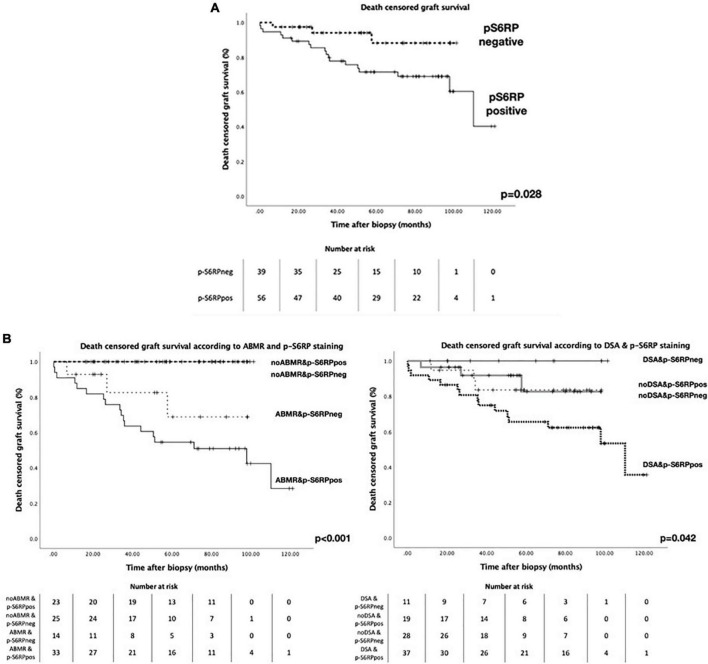
Death censored graft survival in patients with biopsies with p-S6RP positive and negative staining. **(A)** Kaplan-Meier survival curve representing death censored graft survival in patients with positive or negative p-S6RP staining in PTC. **(B Left)**, Kaplan-Meier survival curve representing death censored graft survival in patients with and without ABMR diagnosis according to the p-S6RP staining in the biopsy. *P*-value between ABMR+ cases with positive or negative p-S6RP staining is *p* = 0.21. **(B Right)**, Kaplan-Meier survival curve representing death censored graft survival in patients with and without DSA diagnosis according to the p-S6RP staining in the biopsy. *P*-value between DSA+ cases with positive or negative p-S6RP staining is *p* = 0.043.

We could not find any significant correlation between p-S6RP PTC staining and peritubular capillaritis, PTC multilayering or chronic transplant glomerulopathy (Supplementary Table 2). Interestingly, death-censored graft survival was worse in patients with positive staining of p-S6RP in PTC compared with those with negative staining of p-S6RP (*p* = 0.028, Figure 3A). Nevertheless, considering the impact that ABMR and DSA detection may have in graft survival, we further analyzed graft survival stratifying patients according to the presence or absence of ABMR or DSA, and positive or negative p-S6RP staining (Figure 3B). Our results reflect that worse graft survival is observed in patients with ABMR and positive p-S6RP staining, compared to those with ABMR and negative p-S6RP staining, or without ABMR regardless of p-S6RP staining (*p* < 0.001, Figure 3B). Inferior allograft survival was also observed in cases with DSA and positive p-S6RP staining compared with DSA and negative p-S6RP staining, or non-DSA detection and positive or negative p-S6RP staining cases (*p* = 0.042, Figure 3B).

### mTOR inhibitors immunosuppression did not modulate phospho-mTOR at Ser2448, phospho-S6 ribosomal protein at Ser235/236 or phospho-ERK1/2 (p44/42 MAPK) at Thr202/Tyr204 staining in peritubular capillaries

**FIGURE 4:**
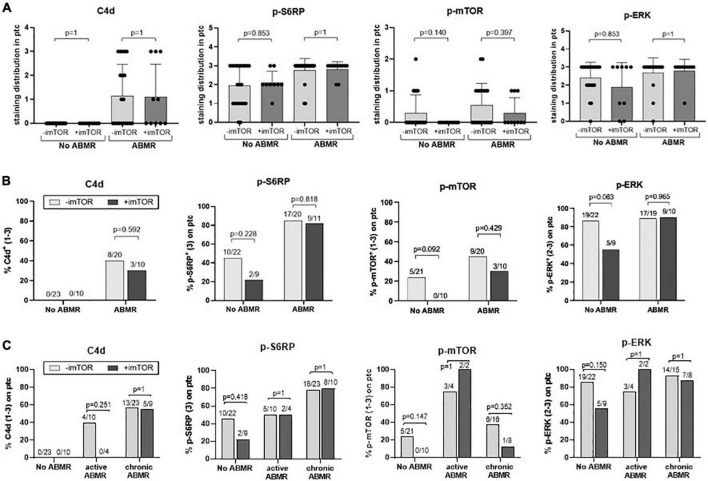
Potential modulation of C4d, p-S6RP, p-mTOR, and p-ERK staining by mTORi immunosuppression. Staining score **(A)** and percentage of positive staining **(B)** of C4d, p-S6RP, p-ERK, and p-mTOR in PTC of renal allografts biopsies with ABMR + DSA+ treated (*n* = 11) or not (*n* = 20) with mTORi, and ABMR–DSA– treated (*n* = 4) or not (*n* = 7) with mTORi. Plots of staining score show the means (bars), standard deviation (error bars), and individual values (dots) for each sample. Kruskal-Wallis test was used to assess significant differences among groups for staining score and Fisher exact test for proportions of staining. **(C)** Percentage of positive staining for C4d, p-S6RP, p-ERK, and p-mTOR in PTC of renal allografts biopsies with active and chronic ABMR, compared to no ABMR, receiving or not mTORi immunosuppression. Fisher exact test was used to assess significant differences among groups.

We evaluated the potential modulation of the mTOR pathway by mTORi immunosuppression. Results showed that treatment with mTORi did not significantly modify C4d staining or phosphorylation of S6RP, ERK or mTOR (Figures 4A,B). Excluding from the analysis four patients treated with mTORi combined with tacrolimus, as their mTORi trough levels were lower, we observed similar results (data not shown). Analysis of mTORi treatment on active (*n* = 14) vs. chronic (*n* = 32) ABMR showed no apparent impact of mTORi on the studied phosphorylated proteins (Figure 4C). These results may suggest that mTORi treatment in our cohort do not modulate p-mTOR, p-S6RP, p-ERK or C4d staining neither in chronic nor in active ABMR, although they need to be confirmed with a higher number of samples.

### Phospho-S6 ribosomal protein at Ser235/236 in peritubular capillaries is associated with human leukocyte antigens class-II donor-specific human leukocyte antigens antibodies

**FIGURE 5:**
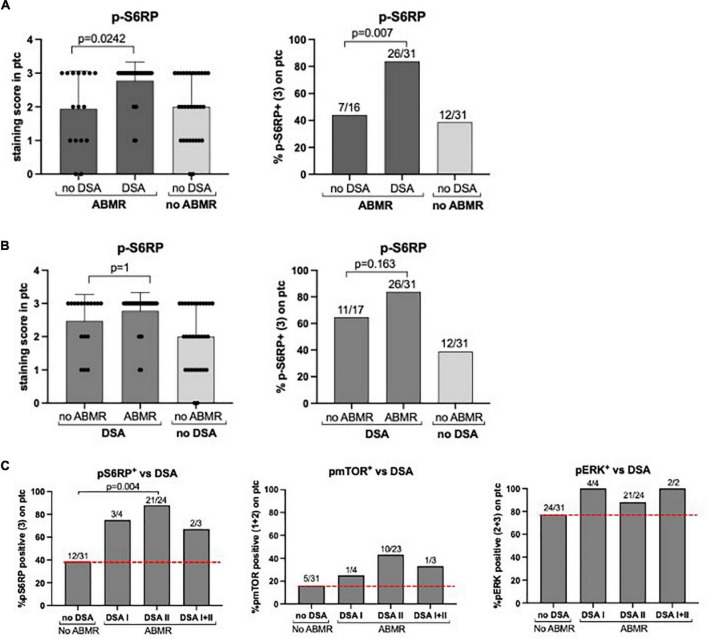
P-S6RP in peritubular capillaries is associated with HLA-DSA. **(A)** Comparison of p-S6RP staining score in biopsies compatible with ABMR with DSA (*n* = 31) and with no detectable HLA-DSA (*n* = 16). **(B)** Comparison of p-S6RP staining score in biopsies with DSA and ABMR (*n* = 31) with biopsies with HLA-DSA but not ABMR (*n* = 18). Plots show the means (bars), standard deviation (error bars), and individual values (dots) for each sample. Mann Whitney test was used to assess significant differences between groups. **(C)** Percentage of positive staining (bars) for p-S6RP, p-mTOR, and p-ERK on biopsies with DSA class I, II, or I&II compared to biopsies with no DSA and no rejection (red dotted line).

Then we assessed whether the positive staining of p-S6RP in ABMR depended upon the presence of circulating HLA-DSA at the time of biopsy. We incorporated a group of patients (*n* = 16) with histological damage in biopsies compatible with ABMR who had no detectable HLA-DSA, and found that p-S6RP was significantly higher in ABMR^+^DSA^+^ compared to ABMR^+^DSA^–^ (*p* = 0.0242, Figure 5A). Next we evaluated whether p-S6RP positivity only occurred in patients with circulating HLA-DSA who had ABMR on their biopsies, and a new group of patients (*n* = 17) with DSA^+^ABMR^–^ was included. There was no significant difference on p-S6RP staining between ABMR^+^DSA^+^ and ABMR^–^DSA^+^ biopsies (*p* = 1, Figure 5B). These results support an association of positive p-S6RP staining with HLA-DSA independently of histological damage.

We also assessed potential differences in p-S6RP staining depending on the type or amount of circulating HLA-DSA. A higher percentage of diffuse p-S6RP staining was observed in ABMR biopsies with DSA class I, II, or I&II compared to no rejection biopsies without DSA (Figure 4C). Considering all four groups of patients, positive p-S6RP staining was associated with total DSA (*p* < 0.001) and with HLA class-II DSA (*p* = 0.002), but not with DSA HLA class-I (*p* = 0.52) (Table 5). The amount of circulating DSA, employing as surrogate the sum of the mean fluorescence intensity of all circulating DSA, did not correlate with p-S6RP staining intensity (Supplementary Figure 2). There was a higher proportion of positive p-mTOR staining biopsies with class-II DSA compared to biopsies with no DSA (Figure 5C) and an association of p-mTOR staining with class-II DSA (*p* = 0.014, Table 5). We found no differences in p-ERK staining comparing different types of DSA with no DSA samples (Figure 5C).

**TABLE 5:** Percentage of positive and negative p-S6RP and p-mTOR staining in peritubular capillaries in biopsy cases with class I, class II, and total DSA.

	p-S6RP+ (*n* = 56)	p-S6RP− (*n* = 39)	*P*-value	p-mTOR+ (*n* = 20)	p-mTOR− (*n* = 44)	*P*-value
Total DSA+	66.1% (37/56)	28.2% (11/39)	**<*0.001***	75.0% (15/20)	40.9% (18/44)	***0.011***
DSA class I+	12.5% (7/56)	7.7% (3/39)	*0.52*	15.0% (3/20)	11.4% (5/44)	*0.70*
DSA class II+	58.9% (33/56)	25.6% (10/39)	***0.002***	70.0% (14/20)	34.1% (15/44)	***0.014***

Five patients had both class I and II HLA-DSA, and have been considered in both groups. A significant *p*-value is indicated by a bold value.

### Combination of phospho-S6 ribosomal protein at Ser235/236 and C4d staining discriminate antibody-mediated rejection with and without donor-specific human leukocyte antigens antibodies

Staining of p-S6RP and C4d did not correlate (*p* = 0.37, Table 6). However, we analyzed whether p-S6RP staining could improve the C4d diagnostic capacity for those cases with ABMR without DSA. Accuracy diagnostics of “C4d,” “p-S6RP,” and “C4d and p-S6RP” for ABMR^+^DSA^+^ and ABMR^+^DSA^–^ are shown through sensitivity and specificity in Table 7, and AUC values for each method were compared in order to determine the best test in terms of diagnostic performance. We found that p-S6RP improves the sensitivity compared to C4d, and that both C4d and p-S6RP together significantly improve the accuracy diagnostics vs. C4d alone, when comparing ABMR with and without HLA-DSA (*p* = 0.017; Table 7). These results indicate that positive p-S6RP staining complements the diagnostic capacity of C4d, which could be helpful for cases with ABMR^+^ DSA^–^.

**TABLE 6:** Correlation table between p-S6RP and C4d staining.

	C4d− (*n* = 78)	C4d+ (*n* = 16)
p-S6RP**−** (*n* = 39)	87.2% (34/39)	12.8% (5/39)
p-S6RP**+** (*n* = 55)	80.0% (44/55)	20.0% (11/55)

Pearson Chi-squared test *p* = 0.42; Pearson correlation p = 0.37.

**TABLE 7:** Diagnostic capability of C4d, p-S6RP, and p-S6RP&C4d to distinguish DSA^+^ from DSA^–^ in cases with ABMR^+^.

	C4d	P-S6RP	P-S6RP and C4d
Sensitivity	38.7%	84.4%	93.8%
Specificity	57.1%	57.1%	42.9%
ROC area	0.479	0.708	0.683
Positive predictive value	80%	90%	88.2%
Negative predictive value	17.4%	44.4%	60%
DeLong test			**0.017** (vs. C4d)

A significant *p*-value is indicated by a bold value.

## Discussion

The diagnostic approach to transplant rejection based on molecular biomarkers may help in the selection of appropriate treatment strategies. We hypothesized that staining of phosphorylated mTOR and its downstream signals S6RP and ERK in kidney allografts may be useful as diagnostic biomarkers and therapeutic targets in ABMR. To test this, we used phosphorylation-specific antibodies to elucidate the effects of anti-HLA antibody-induced signal transduction in PTC endothelium *in vivo*. Our findings support that p-S6RP in peritubular capillary endothelium of kidney graft biopsies is associated with circulating HLA-DSA in the presence or absence of ABMR. In contrast, we could not find a clear significant difference on the phosphorylation of mTOR or ERK comparing ABMR^+^DSA^+^ with ABMR-DSA- biopsies. This could be due to the redundancy of the biologic system in which growth factors and cytokines can also activate PI3K, which in turn phosphorylates mTOR at Ser2448. Furthermore, the phosphorylation of intracellular ERK *in vitro* is described to depend only on mTORC2 activation in the case of HLA class-I DSA (26), and independent of mTOR in the case of HLA class-II DSA (17).

To our knowledge, this is the first study to evaluate the phosphorylation of mTOR proteins in kidney allografts in the context of ABMR. The association between ABMR and the capillary staining of phosphorylated S6K and S6RP was previously reported in heart allograft biopsies (22, 23). Furthermore, class I or class II ligation on *in vitro* endothelial cells results in a time and dose-dependent increase in Ser^235/236^ phosphorylation of S6RP (22). We were expecting to see a correlation between p-S6RP intensity staining and renal damage, however we could not find this correlation, and both active and chronic ABMR presented the same p-S6RP intensity. Instead, we found that high p-S6RP staining was associated with HLA-DSA independently of histological damage.

As novelty, we also had the opportunity to analyze the value of p-S6RP staining in biopsies of KT patients with ABMR without DSA, and in patients with DSA without ABMR. We found that positive staining of p-S6RP was associated with HLA-DSA, specifically with HLA class-II, despite the absence of histological damage compatible with ABMR. Previous work in heart allografts found an association of p-S6RP staining with circulating anti-HLA antibodies, especially also with class II antibodies (22), in patients with cardiac rejection. Interestingly, plasma-derived HLA-DQ antibodies phosphorylate Akt, S6K, and S6RP in microvascular endothelial cells *in vitro* (27). In a clinical setting, we have found that p-S6RP staining evidences the activation of intracellular pathways in the presence of HLA-DSA and could serve as a biomarker of their existence, contributing to ABMR diagnosis. Of note, ABMR without evidence of HLA-DSA showed less frequently p-S6RP in PTC, reflecting that in those cases other triggers could be involved and they may produce damage through other mechanisms. Furthermore, a few cases of ABMR^+^DSA^–^ cases exhibited p-S6RP in PTC potentially due to a low level of HLA DSA undetected with SAB at the time of biopsy (28). In our experience, four ABMR^+^DSA^–^ with diffuse p-S6RP showed detectable DSA later on as shown in Tables 3, 4.

Based on the knowledge of the mTOR pathway and previous *in vitro* studies, we hypothesized that treatment with mTORi in KT patients could modulate the phosphorylation of mTOR and its downstream signals, interfering damage in cases with ABMR. No significant differences in p-S6RP, p-mTOR, p-ERK or C4d staining neither in chronic nor in active ABMR under different immunosuppression were found. These results could suggest that clinical dose and exposure to mTORi may not have been enough in these cases to inhibit the intracellular signaling. The low number of ABMR cases treated with mTORi is one of the limitations of our study, and a higher number of cases would be necessary to confirm these results. Previous *in vitro* studies reported that everolimus effectively inhibited the mTOR activation pathway in anti-HLA class-I antibody-activated endothelial cells (24), pointing to a potential beneficial effect of everolimus in the prevention of chronic ABMR. Of interest, due to the relevance of the mTOR pathway in regulating essential aspects of cell biology, other proteins may have a compensatory activity that allows restoring some of the long-term inhibited mTOR functions. Actually, Ser^235/236^ phosphorylation depends on both p-70S6K and p-90RSK. Therefore, it is conceivable that Ser^235/236^ p-S6RP was not affected by mTORi because it was phosphorylated by p90RSK, an enzyme downstream of ERK not altered by rapalogs. The lack of inhibition of p-ERK by mTORi may be due to compensatory over-activation of ERK in response to antibody-mediated ligation of class II molecules in EC, as previously described (17). Furthermore, the mTOR activity in the kidney has been identified in many different cell types (29) and has been related to ischemia-reperfusion injury (30), interstitial fibrosis (31), and renal carcinoma (32). Therefore, many factors modulate and may impact the mTOR pathway activation in the kidney.

Overall our findings support that p-S6RP in capillary endothelium of kidney graft biopsies is associated with circulating HLA-DSA, independently of the existence of damage in the form of ABMR. These results suggest that p-S6RP staining in kidney allografts evidences the activation of intracellular pathways due to HLA-DSA, and therefore it could serve as a sensitive surrogate biomarker of HLA-DSA for ABMR diagnosis in cases where DSA are not available or not detected yet. p-S6RP emerges as a potential useful tool for prediction of ABMR in the presence of HLA DSA. The lack of correlation of p-S6RP and C4d staining in PTC, despite both being highly associated with circulating HLA DSA and/or ABMR, illustrate two different mechanisms of damage activated by HLA DSA, which deserve different treatment approaches. Larger studies are needed to see if pS6RP could be of interest to assess the value of treatment efficacy in late ABMR in clinical trials.

## Data availability statement

The raw data supporting the conclusion of this article will be made available by the authors, without undue reservation.

## Author contributions

DR-R participated in the design and performance of the research, the data analysis, the figure preparation, and wrote the manuscript. JG participated in the design, performance of the research, and the figure preparation. LL-M participated in acquisition and analysis of the data and the figure preparation. SM, DB, and MR participated in the performance of the research. DR and MP-S participated in acquisition of the data. ER and JP participated in the interpretation of the data. MC participated in the research design, data acquisition, interpretation of results, and wrote the manuscript. All authors contributed to the article and approved the submitted version.

## Supplementary material

Supplementary Figure 1 Death censored graft survival in ABMR + DSA+, ABMR-DSA−, ABMR + DSA−, and ABMR-DSA+ patients. Kaplan-Meier survival curve representing death censored graft survival in the four groups of patients included in the study.

Supplementary Figure 2 Correlation between p-S6RP staining intensity and the MFI of detected DSA.

## Abbreviations

ABMR: antibody mediated rejection
CTG: chronic transplant glomerulopathy
DSA: donor-specific HLA antibodies
eGFR: estimated glomerular filtration rate
HLA: human leukocyte antigens
KT: kidney transplantation
mTOR: mammalian or mechanistic target of rapamycin
mTORi: mTOR inhibitor
p-S6RP: phospho-S6 ribosomal protein at Ser235/236
p-ERK: phospho-ERK1/2 (p44/42 MAPK) at Thr202/Tyr204
p-mTOR: phospho-mTOR at Ser2448
Pr/Cr: urinary protein to creatinine ratio
PTC: peritubular capillaries
TG: transplant glomerulopathy.
