# Supplementary material for: Phosphorylation of S6RP in peritubular capillaries of kidney grafts and circulating HLA donor-specific antibodies
Source: Front Med (Lausanne). 2022 Oct 18;9:988080. doi: 10.3389/fmed.2022.988080 (PMC9622791; doi:10.3389/fmed.2022.988080)
Supplement: Supplementary file 1 [file Data_Sheet_1.docx]

**Supplementary Table 1.** Main demographic and clinical characteristics of patients with ABMR and normal biopsies treated or not with mTOR inhibitors.

|  | **ABMR^+^ DSA^+^ (n=31)** | | | **No ABMR DSA^–^ (n=33)** | | |
| --- | --- | --- | --- | --- | --- | --- |
| **AT BIOPSY** | **Without mTORi**  **(n=20)** | **With mTORi**  **(n=11)** | **P value** | **Without mTORi (n=23)** | **With mTORi**  **(n=10)** | **P value** |
| Biopsy time after KT (months)  [median (p25-p75)] | 77  (14 - 120) | 37  (12 - 56) | 0.13 | 38  (14 - 44) | 37  (21 - 42) | 0.938 |
| Immunosuppression  at biopsy [n (%)]  Prednisone  Calcineurin inhibit.  Mycophenolate | 14 (70)  19 (95)  18 (90) | 11 (100)  2 (18)  9 (82) | 0.066  **<0.001**0.601 | 20 (87)  23 (100)  22 (96) | 10 (100)  6 (60)  4 (40) | 0.536  **0.005**  **0.001** |
| imTOR dose  [median (p25-p75)] |  | 2.5  (1.5 - 4) |  |  | 2.6  (2 - 3) |  |
| imTOR level  [median (p25-p75)] |  | 5.2  (3.3 - 6.8) |  |  | 4.8  (4.6-5.6) |  |
| DSA at biopsy [n (%)]  Negative  Class I only  Class II only  Class I & Class II | 0  3 (15)  16 (80)  1 (5) | 0  1 (9)  8 (73)  2 (18) | 0.658 | 23 (100) | 10(100) |  |
| Graft function  Creatinine [mean (SD)]  eGFR [mean (SD)]  Pr/Cr [median (P25-P75)] | 1.92 (0.91)  42 (20)  676  (184-1079) | 1.71 (0.76)  54 (26)  179  (157-389) | 0.516  0.177  0.060 | 1.32 (0.39)  62 (22)  110  (74-207) | 1.20 (0.47)  68 (32)  128  (77-177) | 0.520  0.909  0.383 |
| Characteristics of ABMR in biopsy [n (%)]  Microinflammation   - glomerulitis ≥ 1 - ptc ≥ 1   C4d deposits in ptc  Chronic TG  PTC multilayering | 17 (85)  16 (76)  15 (71)  8 (38)  10 (48)  8 (38) | 8 (73)  8 (73)  10 (91)  3 (27)  3 (27)  8 (73) | 0.638  0.676  0.383  0.702  0.433  0.136 | 0  0  0  0  0  0 | 0  1  0  0  0  0 |  |

*ABMR: Antibody-mediated rejection, mTORi: mTOR inhibitors, DSA: Donor specific antibodies, SD: standard deviation, KT: kidney transplantation, PTC: peritubular capillaries, TG: transplant glomerulopathy, eGFR: estimated glomerular filtration rate, Pr/Cr: urinary protein to creatinine ratio*

**Supplementary Table 2.** Correlations between p-S6RP staining in PTC and peritubular capillaritis, PTC multilayering or chronic transplant glomerulopathy.

|  | Pearson correlation | Spearman’s rho correlation |
| --- | --- | --- |
| peritubular capillaritis | 0.494 | 0.312 |
| PTC multilayering | 0.471 | 0.343 |
| chronic transplant glomerulopathy | 0.298 | 0.354 |

**Supplementary Table 3.** Comparison between indication and protocol biopsies for the expression of the studied proteins.

|  | Indication biopsy (n=29) | Protocol biopsy (n=68) | Fisher’s exact test |
| --- | --- | --- | --- |
| p-S6RP | 21 (72%) | 35 (51%) | p=0.112 |
| p-mTOR | 11 (38%) | 9 (13%) | **p=0.009** |
| p-ERK | 17 (59%) | 36 (47%) | p=0.709 |
