# Supplementary figures and images for: Phosphorylation of S6RP in peritubular capillaries of kidney grafts and circulating HLA donor-specific antibodies
Source: Front Med (Lausanne). 2022 Oct 18;9:988080. doi: 10.3389/fmed.2022.988080 (PMC9622791; doi:10.3389/fmed.2022.988080)

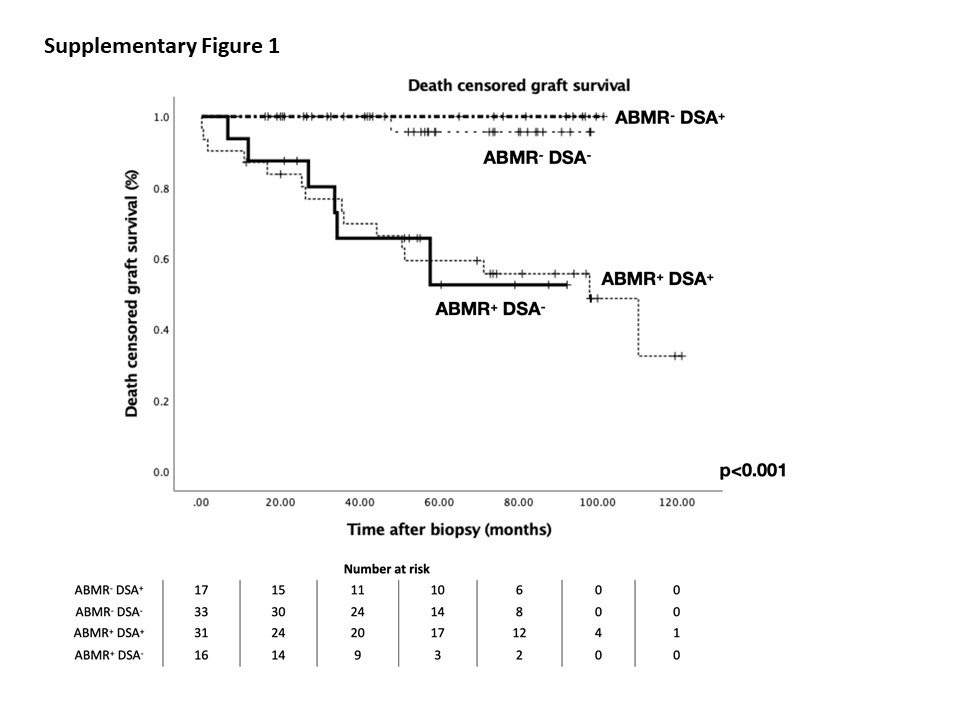

Supplement: Supplementary Figure 1 — Death censored graft survival in ABMR + DSA+, ABMR-DSA−, ABMR + DSA−, and ABMR-DSA+ patients. Kaplan-Meier survival curve representing death censored graft survival in the four groups of patients included in the study. [file Image_1.JPEG]

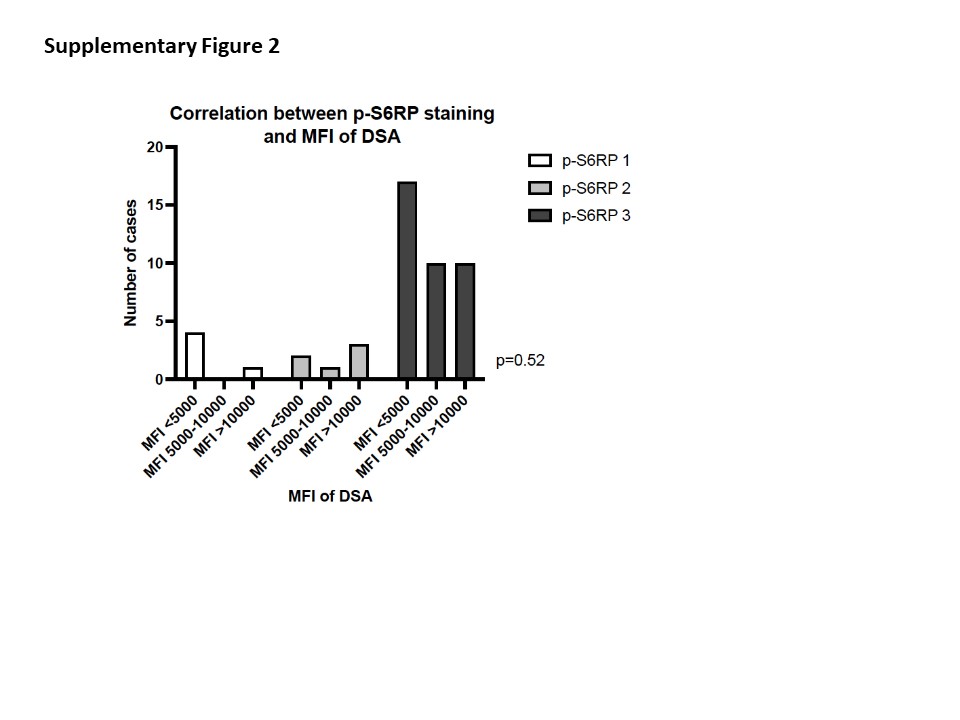

Supplement: Supplementary Figure 2 — Correlation between p-S6RP staining intensity and the MFI of detected DSA. [file Image_2.JPEG]
